# Inconclusive evidence for rapid adaptive evolution

**DOI:** 10.1038/s41467-018-05119-2

**Published:** 2018-07-10

**Authors:** Júlio Manuel Neto, Staffan Bensch, Lars Råberg, Bengt Hansson

**Affiliations:** 1 Department of Biology, Molecular Ecology and Evolution Lab, Ecology Building, SE-223 62 Lund, Sweden; 2 Department of Biology, Functional Zoology, Lund University, Sölvegatan 35, 223 62 Lund, Sweden

Sætre et al.^1^ described a case of decreasing adult body mass in reed warblers (*Acrocephalus scirpaceus*) across a period of 19 years following the restoration of a marshland in Malta. This phenotypic change was interpreted as rapid adaptive evolution because body mass followed a trajectory consistent with that of a population ascending an adaptive peak (a so-called Ornstein–Uhlenbeck model), and correlated with estimates of population fitness and individual survival. Sætre et al.’s^1^ study would thus constitute an example of exceptionally rapid adaptive evolution in the wild. However, we argue that their finding is most likely a result of the inclusion of fat and heavy migrants in the data set, which seriously inflated the average body mass during the first years of the study. Inclusion of migrants can also easily explain the reported mass-related individual survival and population fitness (proportion of birds breeding), as migrants on passage will rarely be recaptured in following years and certainly not breed. Hence, without firm evidence that migrants are excluded from the analyses, we argue that it is premature to infer that the rapid body mass decline is a result of a micro-evolutionary process.

Body mass is a highly variable, plastic trait in birds, both during breeding (e.g. due to development of reproductive organs^2^) and migration (e.g. due to accumulation of fat as flight fuel). In particular, migratory passerines—such as the reed warbler—increase their body mass considerably before and during migration. This can be clearly seen in data from a population in Portugal (Fig. 1; see also results from e.g. Greece^3^ and Spain^4^), which is comparable to Malta as it lies relatively close to the Mediterranean Sea and Sahara Desert. Therefore, to analyse body mass evolution, several factors need to be corrected for, but only diurnal variation was taken into account in Sætre et al.’s^1^ analysis. Their^1^ data set includes birds caught from May to August, which encompasses not only the breeding season, but also the moulting period when the birds replace their body feathers (during which they are known to be heavier^5,6^) and, importantly, also the spring (until mid-June) and autumn (from mid-July) migration periods when individuals from populations breeding north of Malta are passing the island on migration from and towards their African winter quarters. Because the migration period is particularly extensive in this species^7^, it is only during a short period (mid-June to mid-July) when captures of unknown birds can be confidently assigned as local breeders. Hence, it is likely that birds in various conditions (breeding, moulting and migrating) were lumped in Sætre et al.’s^1^ study, which makes any micro-evolutionary interpretation of body mass difficult.

**Fig. 1:**
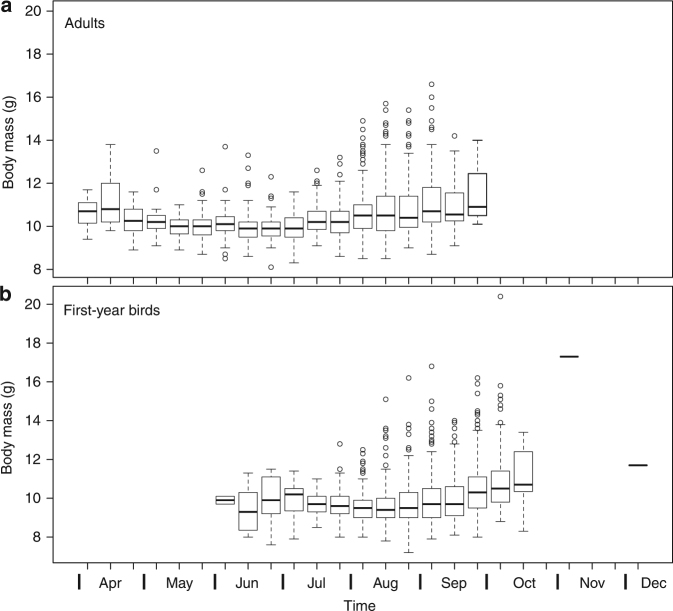
Boxplot depicting the seasonal variation in body mass of **a** adult and **b** first-year reed warblers at Salreu, Estarreja and Portugal, during 1997–2014 (*n* = 1350 adults and 2535 first-years). This site is, as Malta, located in the southern part of the species’ breeding range (c. 550 km north of Malta, a difference that can be covered by migratory reed warblers in 1–3 days flight), but differs in having a very large population of local breeders. The greater body mass of migratory birds caught in April (spring/northward migration) and from August onwards (autumn/southward migration) is obvious despite the fact that (i) only first captures are included here (recaptures show large increases in body mass during the migratory periods); and that (ii) local birds are particularly small (have a low body mass) and abundant, and are breeding almost until the end of August (a period when they are still lean). Indeed, pre-migratory fattening of local birds and the arrival of migrants from the north has been widely described to occur in August at these latitudes^3^. Consequently, between 1 May and 31 August (i.e. during Sætre et al.’s^1^ sampling period), the body mass of both adults and juveniles caught in Portugal varies highly significantly with date (Adults: Mass = 10.29311 − 0.01603Date + 0.00019Date^2^, *F*_2;1193_ = 70.08, *P* = 2.2e−16, df = 1193, *R*^2^ = 0.1051; First-years: Mass = 11.77166 − 0.04875Date + 0.00026Date^2^, *F*_2;1375_ = 8.162, *P* < 0.001, df = 1375, *R*^2^ = 0.01173)

Most importantly, the average body mass in the three first years of Sætre et al.’s study (14.1, 13.4 and 13.1 g) exceeds, to our knowledge, all published average body masses of reed warblers during the breeding season from all across the species distribution, as well as the vast majority of estimates of average body mass during the migratory periods^3,4,8–13^. As an example, the average body mass of adult reed warblers caught during May–August 2013–2016 in a population in southern Sweden (i.e. in the northern range where relatively large birds breed^13^), is only 12.0 g (S.E. = 0.049, *n* = 151; Fig. 2). In fact, the average body mass obtained during the first year of Sætre et al.’s^1^ study even exceeds the mass of the heaviest individual in our sample from southern Sweden (13.6 g, Fig. 2). Since there are no known reed warbler populations with breeding body mass approaching 14 g, it is unclear what the source of the Maltese population could have been (it should be noted that the reed warbler is well studied throughout its range, due to the ease with which it can be mist-netted in reed-beds). Sætre et al^1^. used a sample of reed warblers caught in Malta during September and October as representative of the founder population. We find this puzzling, as this is the period of the year when migratory songbirds attain the greatest body masses after fattening up to prepare for the long migratory flights to cross the Mediterranean Sea and the Sahara Desert^4,9^ (cf. Fig. 1). Furthermore, Sætre et al^1^. consider Italy as the most likely origin of the birds that colonised Malta, but Italian reed warblers have much lower body mass between May and August (mean = 11.06, S.E. = 0.05, *n* = 250 individuals examined in Tuscany, Italy; Francesco Pezzo, in litt.) than the ones reported at the beginning of their study^1^. We argue that the most likely explanation for Sætre et al.’s^1^ result is that migratory birds (with fat reserves) are included in the data set, and that the proportion of such birds was particularly high during the first years of the study. The ratio of migrants to breeders may have been higher in the earlier years of Sætre et al.’s^1^ study, if few birds were breeding in the first years following the colonisation of the locality. Uneven sampling efforts along the season and across years could also generate such a pattern, as could annual variation in the timing of migration and number birds stopping over in Malta (e.g. due to weather). Unfortunately, the sampling design (date and time) was not described in their paper, and the raw data were not made available.

**Fig. 2:**
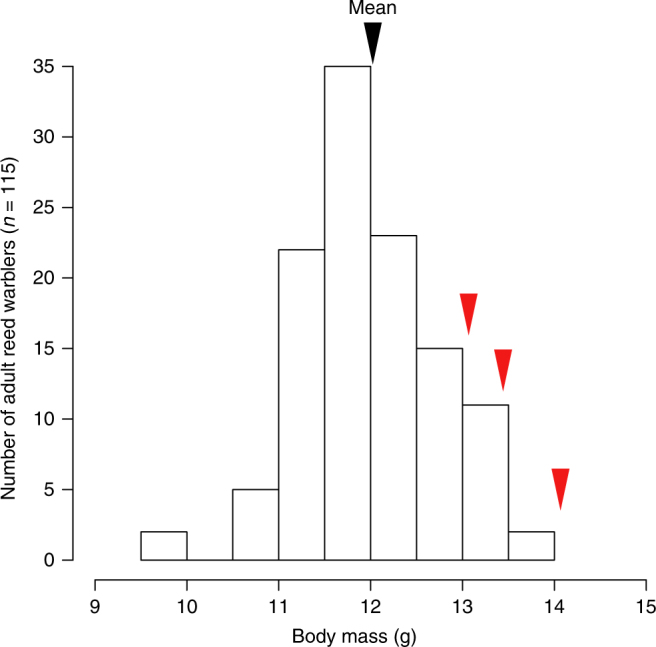
Histogram of body mass of all 115 adult reed warblers in Krankesjön, southern Sweden, during May–August 2013–2016, showing the average for this population (black triangle), and the averages of body mass for the first 3 years of Sætre et al.^1^ study (red triangles). Although Swedish reed warblers are larger than south-European individuals (e.g. compare with data in Fig. 1), the average body mass of the birds caught by Sætre et al.^1^ in Malta during the first 3 years of their study largely exceeds the one for Scandinavian birds, and data from the first year of their study even exceeds the total variation depicted in this histogram. This strongly suggests that a high proportion of migratory birds, which have a large amount of fat reserves, were included in their data set

The presence of migrants in the data set can also explain other results presented by Sætre et al.^1^. In particular, the comparatively low recapture probability (apparent survival) of heavy birds may be caused by these birds being transient individuals migrating through Malta, rather than actually being selected against due to large body mass. The lower population fitness in years where the average body mass was high can also be explained by the presence of a greater proportion of migrants in those years, as ‘fitness’ was measured as the proportion of breeding birds in the data set. Unfortunately, breeders and non-breeders were not distinguished in the study^1^, which makes it impossible to determine whether the small birds were the ones actually reproducing successfully and thus whether selection favoured a decrease in body mass. Furthermore, Sætre et al.’s^1^ support for a high heritability of body mass could have been caused by covariation between age categories in the annual proportion of migrants, since they estimated heritability with a regression between average adult and juvenile body masses per year (i.e. heritability was not based on data of parents and offspring of known relatedness).

In conclusion, we find it very likely that the fit of reed warbler body mass data to an Ornstein–Uhlenbeck model is coincidental, and that Sætre et al.’s^1^ study of rapid evolution in the wild should be considered inconclusive. Because body mass is a highly plastic trait in birds^2^, future studies aiming at describing any potential adaptive response to selection need to use a body mass measure that has been corrected for fat score, and in addition to body mass, report structural size of the birds (such as tarsus, wing and bill lengths).

## Methods

### Sampling

The capture and ringing of birds were conducted under the licenses required by the corresponding national authorities, following standard protocols and releasing the birds unharmed on site. In particular, reed warblers were caught with mist-nets, marked with an aluminium ring issued by the ringing centres of Portugal (CEMPA, ICNF; ringing permits 68/97, 68/98, 45/99, 55/2000, 62/2001, 69/2002, 67/2003, 71/2004, 73/2005, 75/2006, 82/2007, 88/2008, 86/2009, 92/2010, 99/2011, 112/2012, 118/2013) or Sweden (Naturhistoriska riksmuseet, Stockholm; ringing permit 683). Among other measurements, birds were weighed using a digital or a Pesola spring balance to the nearest 0.1 g. In Krankesjön, Skåne, Sweden, 95% of the birds were caught using a constant-effort method, that is, using the same net positions, location and time of day, and during 12 sessions each year evenly distributed from the beginning of May to the end of August; which coincides with Sætre et al.’s^1^ sampling period. Only the first capture of each bird was used to avoid pseudo-replication. The effect of date of capture (1 = 1 May) on body mass was evaluated using a linear regression model in which the quadratic term was included as it lowered the AIC (by more than 10).

### Data availability

The data are available in Supplementary Data 1.

## Electronic supplementary material

Description of Additional Supplementary Files

Supplementary Data 1
