# Supplementary material for: Inconclusive evidence for rapid adaptive evolution
Source: Nat Commun. 2018 Jul 10;9:2663. doi: 10.1038/s41467-018-05119-2 (PMC6039431; doi:10.1038/s41467-018-05119-2)
Supplement: Supplementary file 1 — Description of Additional Supplementary Files [file 41467_2018_5119_MOESM1_ESM.pdf]

Description of Additional Supplementary Files

File Name: Supplementary Data 1

Description: Raw data used to produce Figures 1 and 2. It includes the data obtained during the first capture of reed warblers in Portugal and in Sweden, especially, the site, date, age and weight of each individual; complemented by biometrics, fat and muscle scores and other additional information gathered during the fieldwork.
